# Quasi-band structure of quantum-confined nanocrystals

**DOI:** 10.1038/s41598-023-31989-8

**Published:** 2023-03-22

**Authors:** Marius Buerkle, Mickaël Lozac’h, Davide Mariotti, Vladimir Švrček

**Affiliations:** 1 National Institute of Advanced Industrial Science and Technology (AIST), Tsukuba, Japan; 2 Integrated Bio-Engineering Centre (NIBEC), University of Ulster, Coleraine, UK

**Keywords:** Density functional theory, Electronic properties and materials

## Abstract

We discuss the electronic properties of quantum-confined nanocrystals. In particular, we show how, starting from the discrete molecular states of small nanocrystals, an approximate band structure (quasi-band structure) emerges with increasing particle size. Finite temperature is found to broaden the discrete states in energy space forming even for nanocrystals in the quantum-confinement regime quasi-continuous bands in k-space. This bands can be, to a certain extend, interpreted along the lines of standard band structure theory, while taking also finite size and surface effects into account. We discuss this on various prototypical nanocrystal systems.

## Introduction

Nanocrystals (NC) with sizes comparable to their exciton Bohr radius^1–5^ can have very unique electronic properties arsing from the interplay between quantum-confinement, finite-size effects, and surface functionalization. The degree of quantum-confinement, which can be for example controlled by the particle diameter as well as different functional groups on the NC’s surface, allows to tune electronic and optical properties, such as the electronic gap, absorption and luminescence^6–11^. Nanocrystals with tailored properties are very attractive for various application requiring distinct optical and electronic properties, such as fluorescent NC for bio-imaging, light absorbing NC for next-generation photovoltaics or luminescent NC for light emitting devices^9,12–18^.

Essentially, quantum-confined NCs are systems that fall somewhere in between the molecular and the bulk world. On the one hand NC have often a well-defined crystal structure, which remains close to their bulk counterpart and on the other hand the finite surface can have a large effect on the electronic and optical properties. Concepts from both worlds are often used loosely and interchangeably. For example the electronic gap of NCs is usually termed band-gap rather then HOMO–LUMO gap, i.e. the electronic gap between the highest occupied and lowest unoccupied (discrete) molecular orbital, moreover the nature of the band gap is often labeled to be direct or indirect following standard semiconductor theory. These are concepts which are only strictly valid for bulk crystals with a well-defined band structure^19^ and do not necessarily correspond to properties of finite-sized systems with broken translational symmetry and discrete energy levels. However, it would be desirable to connect the intuitive picture of band structure theory with nanocrystals while also accounting for finite-size and surface effects. The transition from molecular regime to quantum-confined NCs has been discussed in terms of surface- and core-states^20^, exciton interaction^21^, and break-down of the effective mass description.^22^ In this work we are going to show how an approximate band structure^10,23^ (quasi-band structure) can provide a simple and intuitive way to describe nanocrystals. The quasi-band structure stays close to standard band structure theory, while taking finite-size and surface effects into account. In particular, we discuss the effects of (i) size and (ii) surface functionalization on the electronic structure of silicon nanocrystals and (iii) how an approximate band structure emerges from the discrete molecular orbitals with increasing particles size and finite temperature. This is discussed for metallic NCs and semiconducting NCs with indirect and direct band-gap.

## Methods

### Quasi-band structure

The band structure of bulk crystals is a direct consequence of their translational symmetry and follows from Bloch’s theorem^19,24^. Concepts from band structure theory, such as band gap, valence band maximum (VBM), or conduction band minimum (CBM) are often used in the context of nanocrystals, however for nanocrystals, as finite-sized objects, they are not (strictly) valid. In particular, quantum confined nanocrystals, while their size is usual much bigger than molecules they are still well-below the bulk limit. On the other hand, NCs have usually a well-defined crystal structure which converges inside the NC to values close to bulk^10^. To connect the molecular limit with the bulk limit we will construct an approximate band structure (quasi-band structure) by considering the k-space resolved density of states (DOS) for each (molecular) state^5,10,23,25^, which is closely related to angle-resolved photoemission spectroscopy^26^. By doing so we take surface and finite size into account, while staying as close as possible to the intuitive concept of the standard band structure theory. Following references^10,27^ the DOS is obtained using a Green’s functions technique, where all relevant quantities can be obtained from first-principles calculations. The k-space resolved DOS is obtained from the spectral representation of the Green’s function^28^

1 $$\begin{aligned} {\hat{G}}=\sum _{\mu }\frac{\left| \mu \right\rangle \left\langle \mu \right| }{E+\text {i}\eta -\epsilon _{\mu }}, \end{aligned}$$

here $\left| \mu \right\rangle$ is an energy eigenstate of Hamiltonian ${\hat{H}}$ to the eigenvalue$\epsilon _{\mu }$ and $\eta >0$ is an infinitesimal quantity. Keeping $\eta$ as a phenomenological parameter finite would allow to account for the finite lifetime (broadening) of the discrete energy eigenstates or more rigorously interaction effects could introduced by including appropriate self-energy terms into Eq. (1). Here, we will neglect interactions, taking only the intrinsic thermal broadening of the DOS into account. From Eq. (1) we can directly calculate the (local) DOS $\rho _{\mu }\left( E\right) =-\frac{1}{\pi }{ \rm Im}\left( G_{\mu \mu }\left( E\right) \right)$^28^. An approximate representation in k-space is obtained by projecting the molecular states in real space $\phi _{\mu }\left( \vec {r}\right) =\left\langle \vec {r}|\mu \right\rangle$ onto plane waves $\phi _{\mu }\left( \vec {k}\right) =\left\langle \vec {k}|\mu \right\rangle =\int {\rm d}\vec {r}\left\langle \vec {k}|\vec {r}\right\rangle \left\langle \vec {r}|\mu \right\rangle =\int \text {d}\vec {r}e^{{\rm i}\vec {k}\cdot \vec {r}}\phi _{\mu }\left( \vec {r}\right)$^23^. This allows to write Eq. (1) as

2 $$\begin{aligned} G\left( E,\epsilon _{\mu };\vec {k}\right) =\sum _{\mu }\frac{\phi _{\mu }^{*}\left( \vec {r}\right) \phi _{\mu } \left( \vec {r}\right) }{E+{\rm i}\eta -\epsilon _{\mu }} \end{aligned}$$

and to obtained the k-space resolved DOS of state $\mu$ at temperature $T=0\ K$ from

3 $$\begin{aligned} \rho \left( E,\epsilon _{\mu };\vec {k}\right) =-\frac{1}{\pi }{ \rm Im} \left[ G\left( E,\epsilon _{\mu };\vec {k}\right) \right] . \end{aligned}$$

Whereas off-diagonal terms in k-space arising due to the broken translational symmetry are neglected^29^. The thermodynamic density of states $N\left( E,T\right) =\partial n\left( E,T\right) /\partial E$ at a given finite temperature *T* is related to the DOS at $T=0\ K$ ( Eq. 1) via the electron density $n\left( E,T\right) =\int _{-\infty }^{\infty }{{\rm d}E_{1}\rho \left( E_{1}\right) f\left( E_{1}-E,T\right) }$. Hence, the temperature dependent k-space resolved DOS is given by the convolution of the DOS at $T=0\ K$ (Eq. 3) with the energy derivative of the Fermi function $f^{\prime }\left( E-E_{1},T\right) =-\partial f\left( E-E_{1},T\right) /\partial E$

4 $$\begin{aligned} N\left( E,T;\vec {k}\right) =&\sum _{\mu }\int _{-\infty }^{\infty }\text {d}E_{1}\rho \left( E_{1},\epsilon _{\mu };\vec {k}\right) \\&\times f^{\prime }\left( E-E_{1},T\right) .\nonumber \end{aligned}$$

As we will see in the following the thermal broadening will lead, even for small nanocrystals, to the formation of quasi-continuous bands-like features.

**Figure 1:**
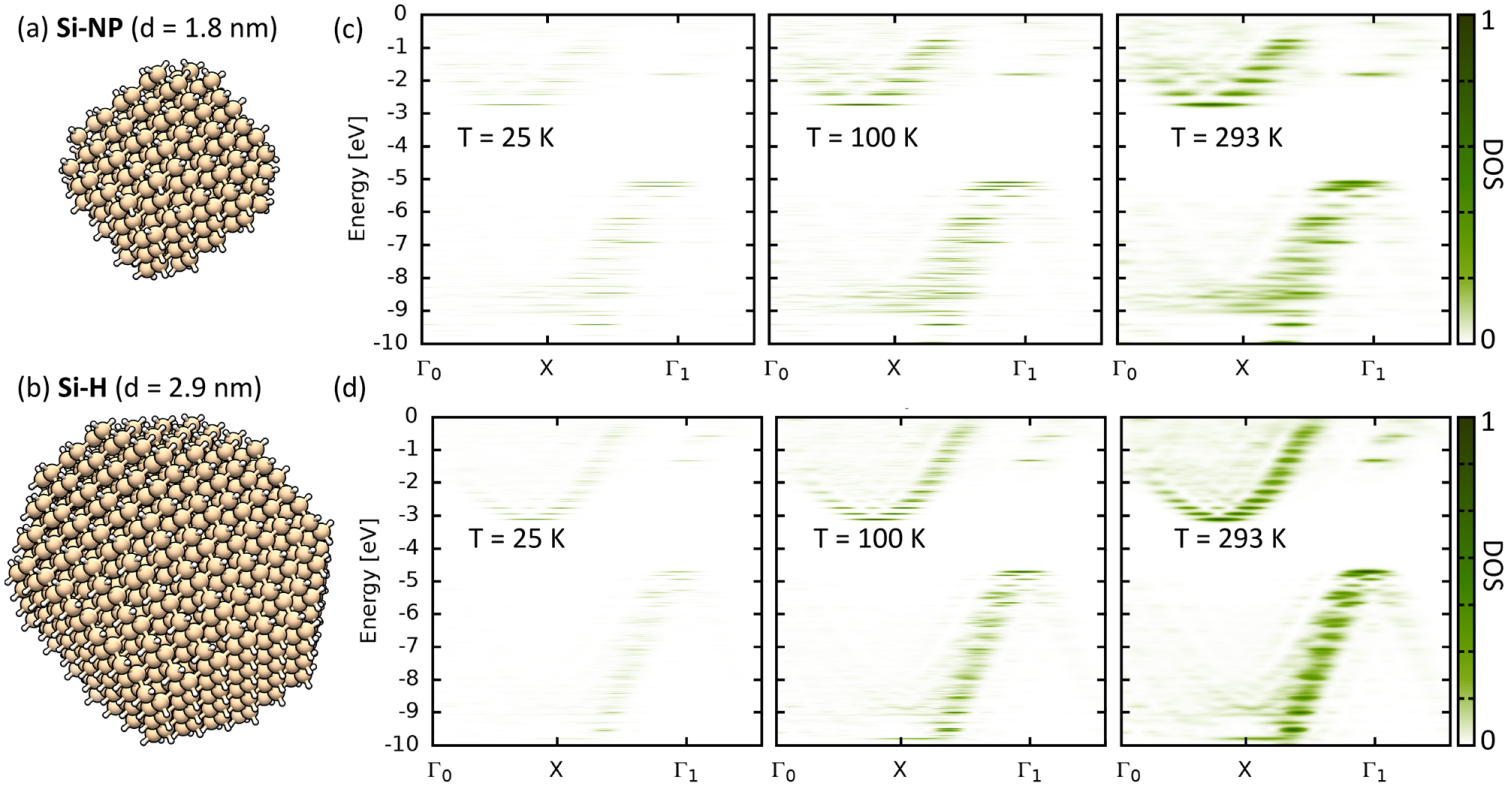
Geometry of the Si-H nanocrystal with an approximate diameter of (**a**) $\text {d}=1.8\,\text {nm}$ and (**b**) $\text {d}=2.9\,\text {nm}.$ Quasi-band structure calculated at different temperatures *T* for (**c**) Si-H ($\text {d}=1.8\,\text {nm}$) and (**d**) Si-H ($\text {d}=2.9\,\text {nm}$).

### First-principles calculations

The electronic and geometrical properties of the NC were obtained within density functional theory (DFT) using the PBE functional^30^ and a double-zeta basis set with polarization functions for all nonhydrogen atoms^31,32^. The total energies were converged with a precision of $10^{-7}\ \text {a.u.}$. The structures of all NC were fully relaxed, and the optimization was carried out until the maximum norm of the gradient drops below $10^{-5}\ \text {a.u.}$. The quantum chemistry package TURBOMOLE^33^ was used for all calculations.

To model the nanocrystal geometry we generate an initial structure with a certain diameter cutout from the corresponding ideal bulk lattice with under-coordinated surface atoms removed and subsequently fully relaxed. The initial lattice parameters are given by, Si face centered cubic ($a=5.43\,{\mathring{\text {A}}}$)^34^, GaAs face centered cubic ($a=5.63\,{\mathring{\text {A}}}$)^34^, Pb face centered cubic ($a=4.95\,{\mathring{\text {A}}}$)^34^, PbSe face centered cubic ($a=6.12\,{\mathring{\text {A}}}$)^35^, and CdSe face centered cubic ($a=5.43\,{\mathring{\text {A}}}$)^34^.

For the semiconducting Si and GaAs NCs the surface is fully passivated with hydrogen atoms, for the metallic Pb NC and PbSe we assume a pristine surface without oxidation as observed in recent experiments^36,37^. For CdSe NCs we consider pristine as well as OH functionalized surfaces.

**Figure 2:**
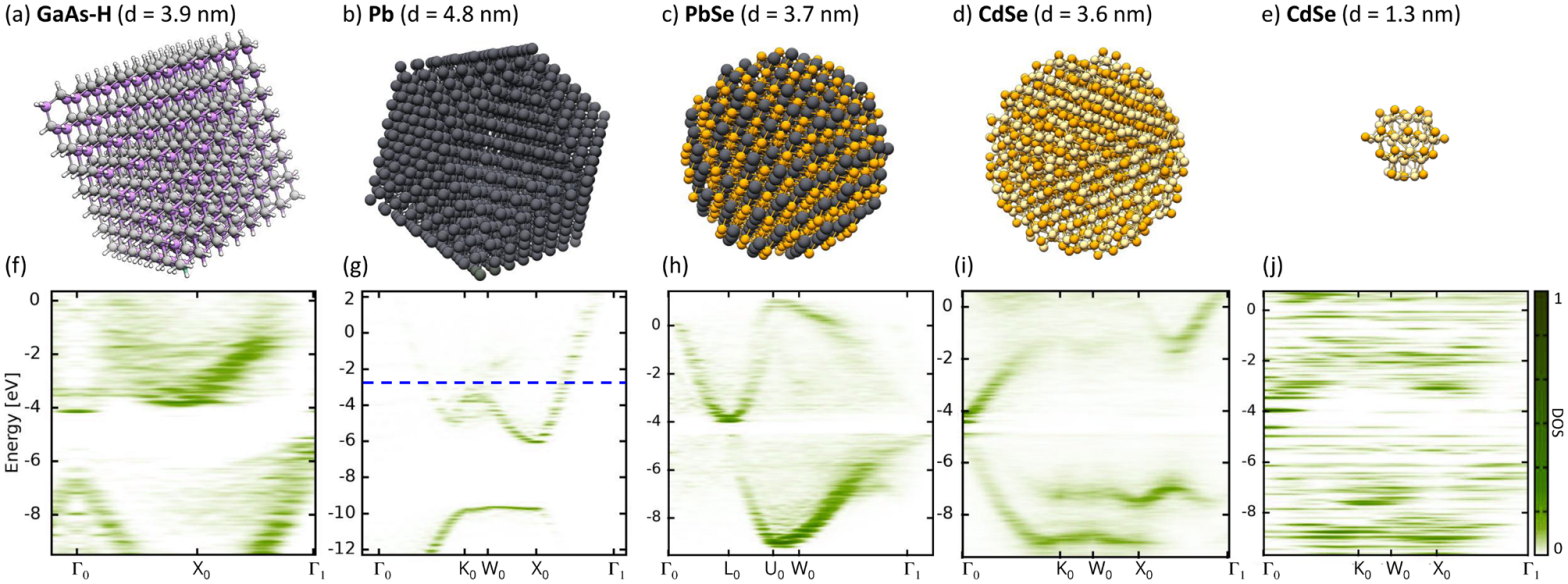
Geometry of (**a**) the GaAs nanocrystal, (**b**) the Pb NC, (**c**) the PbSe NC, (**d**) the CdSe NC, and (**e**) ultra-small CdSe NC. The corresponding quasi-band structures are given in (**f**) for GaAs, (**g**) for Pb, (**h**) PbSe, (**i**) CdSe, and ultra-small CdSe. The dotted blue line indicates the Fermi energy and atoms are given as color coded spheres, Ga (gray), As (purple), H (white), Pb (black), Se (orange), and Cd (yellow).

## Results and discussions

### Quasi-band structure, size and temperature dependence

First we want to investigate how the quasi-band structure emerges with increasing particle size and how the bands are formed due to finite temperature. Whereas, we purely focus on temperature effects as described by Eq. (4), i.e., a smearing of the occupation following the Fermi distribution, renormalization of the orbital energies and finite lifetimes of the molecular states due to e.g. electron-phonon interaction is neglected here, however it would be possible to account for such effects by introducing the corresponding self-energies to the Green’s functions which are used to calculated the DOS.

In the following the symmetry points are given with respect to their bulk values, while the lattice parameter varies for fully relaxed NCs the deviations are found to be small for all NCs studied here. We start with a silicon nanocrystal (Si-NC) with very small diameter of $d=1.8\ \text {nm}$ (Fig. 1a), as here the discrete nature of the energy levels should be clearly evident. The corresponding quasi-band structure for different temperatures is given in Fig. 1c. The total number of energy eigenstates is limited for the small Si-NC ($d=1.8\ \text {nm}$) and their broadening remains small at low temperature ($T=25\ \text {K}$) however, while the discrete nature of the eigenvalues is still clearly visible, it is already possible to see the emergence of band-like features. Increasing the temperature broadens the energy levels, and due to the finite overlap between states close in energy, quasi continuous bands start to form. At room temperature ($T=293\ \text {K}$) the quasi continuous bands are clearly visible, yet small sub gaps within the bands remain even at room temperature. Increasing the particle size to $d=2.9\ \text {nm}$ (Fig. 1b) increasing the number of molecular states, which tends to close the sub gaps, as the states become more and more dense in energy space (Fig. 1d). Additionally the bands become more localized in k-space as the surface effects decrease. Lastly, it must be stressed that due to the broken translational symmetry k-points in different Brillouin zones are not equivalent. While for $T=293\ \text {K}$ the quasi-band structure shows similar feature as the bulk one, i.e. approximate shape of the bands and position of the valance band minimum (X) and conduction band maximum ($\Gamma$), the two $\Gamma$-points in adjacent reciprocal cells, $\Gamma _{0}$ and $\Gamma _{1}$ are not equivalent. The bands are more pronounced towards $\Gamma _{1}$ while the weight at $\Gamma _{0}$ remains smaller. Nonetheless we clearly see that Si-NC are just as their bulk counter part an indirect band gap materials with a transition between $\Gamma$ and *X* point, or to be precise between one of the distinct $\Gamma$ and one of the *X* points.

More examples for the quasi bandstructure of a semiconducting NC with a direct band gap and a metallic NC, namely a hydrogen terminated GaAs-NC with $d=3.9\,\text {nm}$ (Fig. 2a) and a Pb-NC with $d=4.9\,\text {nm}$ (Fig. 2b) are given in Fig. 2f and 2g. The quasi-band structure is calculated at room temperature $(T=293\,\text {K})$. Bulk GaAs is direct band gap material, similarly the GaAs-NC shows also a direct transition at the $\Gamma$ points. While the weight between the two non-equivalent points $\Gamma$ varies, the overall shape of the bands remains similar. Additionally, small sub gaps are present at the $\Gamma$ points (Fig. 2f), they can likely be attributed to localized surface states.

Bulk Pb is a metal, thus has no band gap and a finite DOS at the Fermi energy. For Pb-NC localization introduced due by the finite NC surface leads naturally to an, albeit possibly small, electronic gap. The HOMO-LUMO gap of the Pb-NC at $T=0\,\text {K }$is only $0.05\,\text {eV}$, which is consistent with very small energy gaps observed for Pb NCs at low temperatures^38^. If we follow the Aufbau principle and assume a Fermi smearing of the occupation we get a broken occupation around the HOMO-LUMO gap and can calculated an effective Fermi-energy which is indicated by the dotted blue line in the quasi-band structure in Fig. 2g. The bands for the Pb-NC are already well defined as the particle size is relatively big. Accordingly, the Pb-NC is effectively metallic at room temperature. If we move away from the Fermi energy to lower energies we get similarly to bulk Pb a gap in the band structure with a flat band connecting the K and X point.

Another prominent class of nanoparticle are metal-selenide NCs^39^. Here we consider lead selenide and cadmium selenide, PbSe-NC with $d=3.7\,\text {nm}$ (Fig. 2c) and CdSe-NC with $d=3.6\,\text {nm}$ (Fig. 2d). For both NC the quasi bandstructure reproduces the main features of the bulk bandstructure. The PbSe-NC has a direct band gap with the CBM and VBM like features situated at the L point (Fig. 2h), which is consistent with the corresponding bulk bandstructure of PbSe^40^ as well as what was observed from Auger recombination measurments in PbSe nanocrystals^41^. The CdSe-NC has a direct band gap located at the $\Gamma$ (Fig. 2i), which is also consistent with experimental observations^41^. We also observe gap states which are likely induced due to the rather strong surface reconstruction in the case of CdSe-NC, for all other studied NC the surface reconstruction remained small. Besides the features around the band gap, the quasi bandstructure also shows the flat-band between the K point and X point Fig. 2i), showing a comparable behavior to the bulk bandstructure^40^. For CdSe ultra-small NCs can have diameters in the order of $1\,\text {nm}$^20^. The quasi bandstructure for a CdSe-NC in this regime, with $d=1.3\,\text {nm}$ (Fig. 2e), is given in Fig. 2j. The localization of features in k-space have largely disappeared and it is not possible to identify band-like features, which is consistent with what has been suggested for CdSe-NC in the molecular cluster regime^20^.

### Surface functionalization

**Figure 3:**
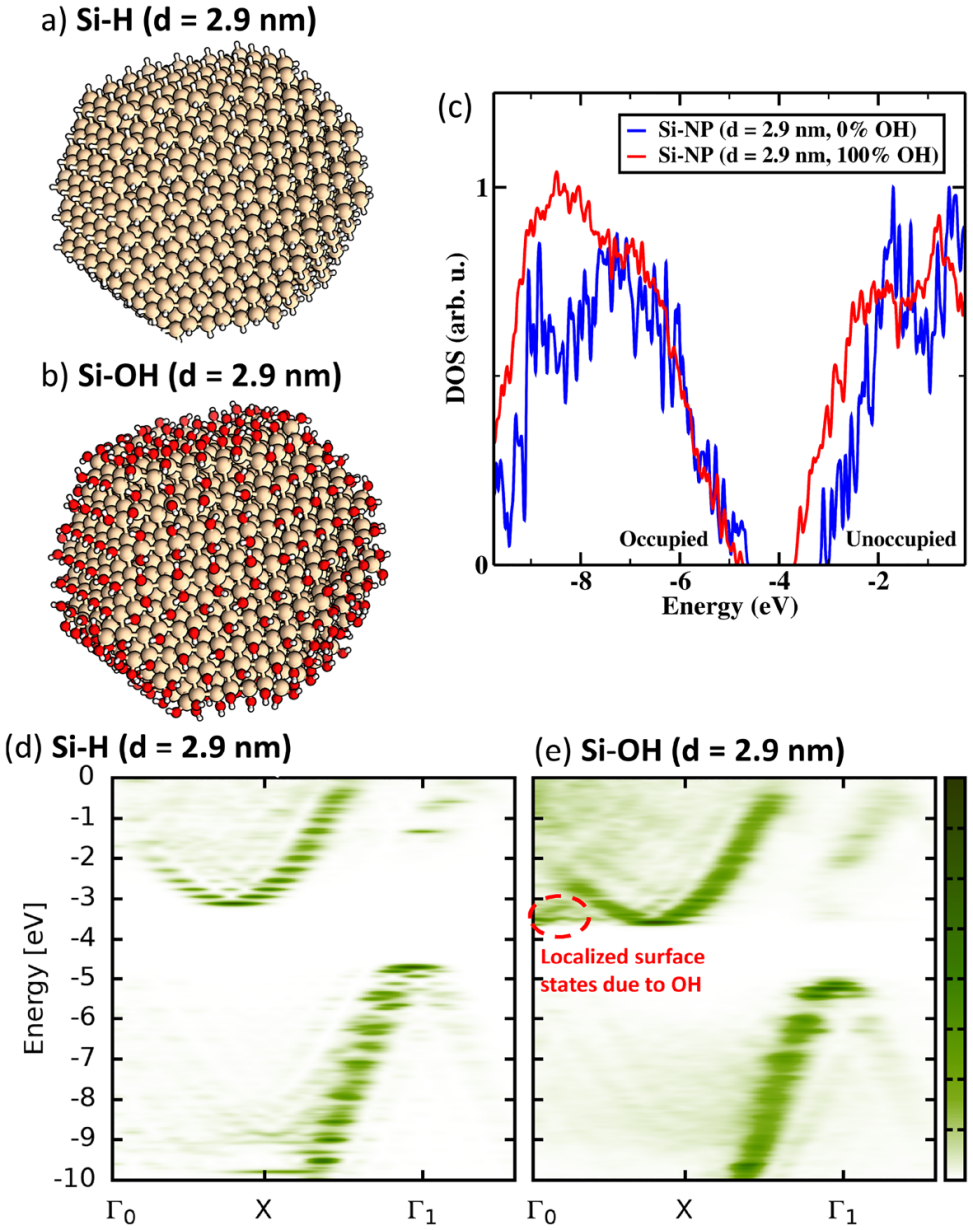
Density of states for pristine Si-NC with 0% OH coverage (blue) and Si-NC with full 100% OH coverage (red) for (**a**) $d=1.8\ \text {nm}$ and (**b**) $d=2.9\ \text {nm}$. The DOS is calculated by Gaussian broadening (0.025 eV) and superimposing each discrete molecular energy level.

In the following we consider approximate spherical silicon nanocrystals with varying degree of hydroxyl (OH) surface coverage The modeling here follows our previous theoretical and experimental works^8,10^. Using plasma processing techniques, it is not only possible to synthesize high quality NC with a narrow size distribution and well-defined crystal structure but also to control the degree of surface functionalization^8,9^. Depending on the processing time it is possible to replace the initially hydrogen termination of the NC surface (Fig. 3a) gradually with OH groups until the NC surface is completely functionalized by hydroxyl (Fig. 3b)^10^.

**Figure 4:**
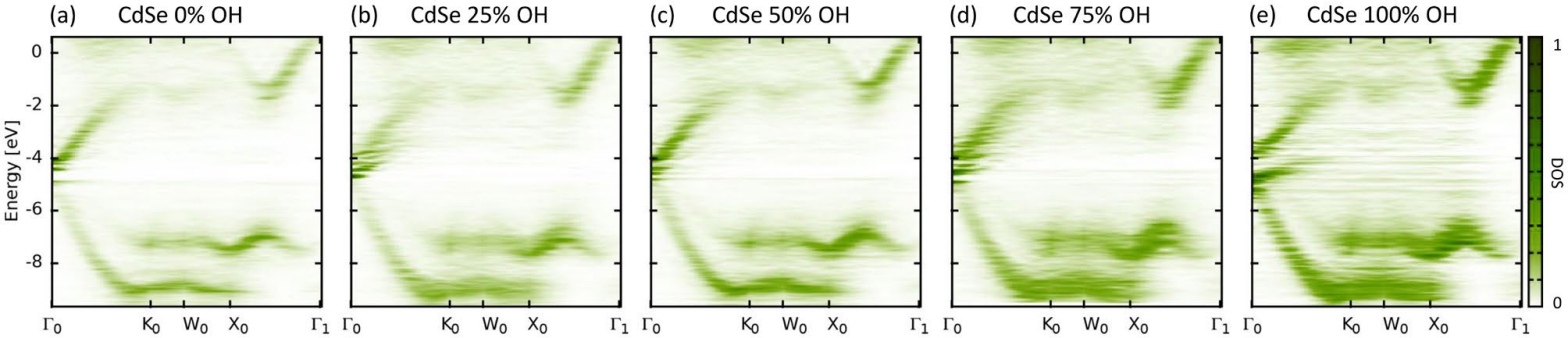
Quasi-band structures of CdSe NCs with (**a**) 0% OH coverage, (**b**) 25% OH coverage, (**c**) 50% OH coverage, (**d**) 75% OH coverage, and (**e**) 100% OH coverage.

Surface functionalization can have several distinct effects on the NC, (i) chemical effects due to e.g. charge transfer, (ii) change of structural properties due to strain, and (iii) induce localized surface states. Here, charge transfer between the electron withdrawing hydroxyl groups and the silicon atoms of the outer silicon shells plays the main role, while the induced strain remains small and has only minor influence on the electronic properties^10^. In Fig. 3c we compare the DOS of Si-NC with full hydrogen coverage to the DOS of the Si-NC with full OH coverage. Introducing hydroxyl on the Si-NC surface has two effects on the DOS it leads to a smearing of the overall DOS and due to the electron donating nature of OH to a shift the orbitals to lower energies, whereas the effect is more pronounced for the unoccupied frontier orbitals which tend to be more localized at the NC’s surface. The overall features of the DOS translate accordingly to the quasi-band structure. That is replacing the hydrogen atoms with OH leads, as already suggested from the joined DOS, to a smearing of the bands making them overall more “fuzzy” and more delocalized in k-space (Fig. 3d,e). The energy as well as the shape of the occupied bands remain otherwise largely unchanged as the occupied frontier orbitals tend to be localized within the NC. On the other hand, the unoccupied frontier orbitals tend to be localized on the outer shells of the Si-NC and thus the influence of the surface functionalization is larger on unoccupied states. Accordingly, the unoccupied states are moved down in energy. The energy of lowest unoccupied molecular orbital (LUMO) decreases by around 0.8 eV if the hydrogen coverage is fully replaced by OH groups. This reduction of the electronic gap is consistent with the experimentally observed red shift of the absorbance spectrum with increasing OH coverage^10^. Moreover, additional flat bands in conductance band due to localized surface states are formed at the $\Gamma$ point (Fig. 3e).

Hydroxyl is also often used as precursor for more complex surface groups attached to CdSe NCs^42^. For CdSe, similar to Si NCs, the overall features of the band-structure remain stable as compared to the pristine NC (Fig. 4a), however due to the increased surface reconstruction the bands become much broader with increased OH coverage (Fig. 4b–e). The main influence of the hydroxyl groups is the formation of states within the bandgap, while states further away from gap remain largely unaffected other then the aforementioned broadening. With increasing OH coverage this gives rise to the formation of sub-bands which are mainly localized around the $\Gamma$ point. For high OH coverage (Fig. 4e) this can even results in quasi-metallic NCs.

## Conclusion

We have introduced the concept of a quasi-band structure which allows us to get an approximate band-like description for objects with sizes falling in between finite-sized molecules and bulk materials. The quasi-band structure allows us to apply, at least approximately, many concepts from standard band structure theory, while at the same time account for finite-size effects. We applied this approach to quantum confined nanocrystals for which surface effects are particular important. Even for small nanocrystals band-like features emerge due to the broadening of the energy eigenstates at finite temperatures. With increasing particle size the band-like features become more and more pronounced. We showed that it is possible to identify clearly the direct and indirect characteristics of band gaps as well as connect the quasi-band structures to features of the corresponding bulk material. Also effects induced by surface functionalization can be readily identified and characterized by means of the quasi-band structure.

## Data Availability

The data and atomic structures are available from the corresponding author on reasonable request.
